# Supplementary material for: Replicate Testing of Clinical Endpoints Can Prevent No-Go Decisions for Beneficial Vaccines
Source: Vaccines (Basel). 2023 Sep 19;11(9):1501. doi: 10.3390/vaccines11091501 (PMC10535203; doi:10.3390/vaccines11091501)
Supplement: Supplementary file 1 [file vaccines-11-01501-s001.zip › Supplement/vaccines-2459398-Supplementary Text.pdf]

Supplementary Materials:

Replicate testing of clinical endpoints can prevent no-go decisions for beneficial vaccines

Daniel I. S. Rosenbloom, Julie Dudášová, Casey Davis, Radha A. Railkar, Nitin Mehrotra and Jeffrey R. Sachs

Section S1. Claim about increasing the number of replicates:

Here we prove that for a majority rule strategy with odd *n*, the effective FP (equivalently, FN) rate decreases with *n*, for any positive FP (equivalently, FN) rate less than 0.5. As noted in Section 2.3, the expressions for effective FP and effective FN rates are equivalent, substituting in the appropriate rate. So it suffices to show that the effective FP rate decreases as the number of replicates increases from *n* to *n* + 2.

Suppose that *n* replicate assays are performed on a non-infected sample. Let $m = (n+1)/2$, the majority count. There are two cases where the outcome changes after adding two more replicates:

Case #	Number of FPs (out of *n*)	Probability of this case (for *n* replicates)	Outcome of majority rule strategy	Requirement for two more replicates to change outcome	Probability of change
1	$m-1$	$\binom{n}{m-1}\mathrm{FP}^{m-1}(1-\mathrm{FP})^{n-m+1}$	No FP	Both must be FP	FP^2
2	m	$\binom{n}{m}\mathrm{FP}^{m}(1-\mathrm{FP})^{n-m}$	FP	Bot must *not* be FP	$(1-\mathrm{FP})^2$

As the number of replicates increases from *n* to *n* + 2, the effective FP rate changes by an amount equal to the difference in the change probabilities from this table:

P(switch from negative to positive) – P(switch from positive to negative)

$$= \mathrm{FP}^2\binom{n}{m-1}\mathrm{FP}^{m-1}(1-\mathrm{FP})^{n-m+1} - (1-\mathrm{FP})^2\binom{n}{m}\mathrm{FP}^{m}(1-\mathrm{FP})^{n-m}$$

The claim that the effective probability decreases with *n* is therefore equivalent to the inequality,

$$\frac{(1-\mathrm{FP})^2\binom{n}{m}\mathrm{FP}^{m}(1-\mathrm{FP})^{n-m}}{\mathrm{FP}^2\binom{n}{m-1}\mathrm{FP}^{m-1}(1-\mathrm{FP})^{n-m+1}} > 1$$

The left hand side simplifies:

$$\frac{\binom{n}{m}\mathrm{FP}^{m}(1-\mathrm{FP})^{n-m+2}}{\binom{n}{m-1}\mathrm{FP}^{m+1}(1-\mathrm{FP})^{n-m+1}}$$

$$= \frac{\binom{n}{m}(1-\mathrm{FP})}{\binom{n}{m-1}\mathrm{FP}}$$

Since $m = (n+1)/2$, the two binomial coefficients are equal, and the ratio is $(1-\mathrm{FP})/\mathrm{FP}$, which exceeds 1 precisely for FP < 0.5.

Supplementary Figures

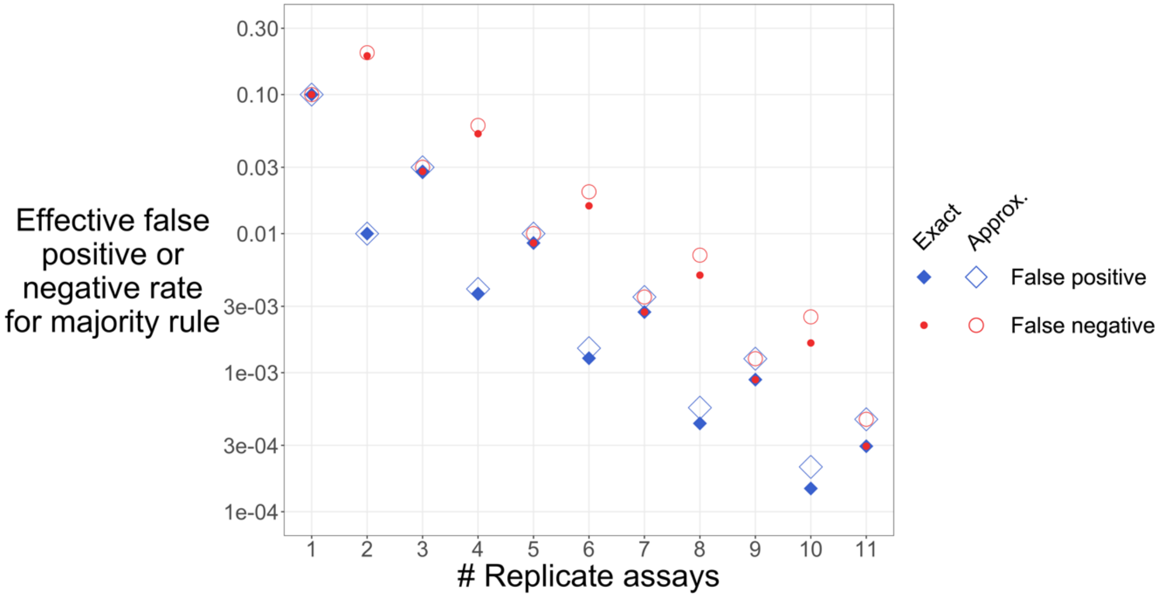

Figure S1. Adequacy of approximation for majority rule sampling strategy. Filled symbols show actual values (Eqs. 9 and 10, Methods); open symbols show respective approximations (Eqs. 11 and 12, Methods). As noted in Section 2.3, for odd numbers of replicate assays, computed values of effective false positive and false negative rates are the same. **Parameters:** FP = FN = 0.1.

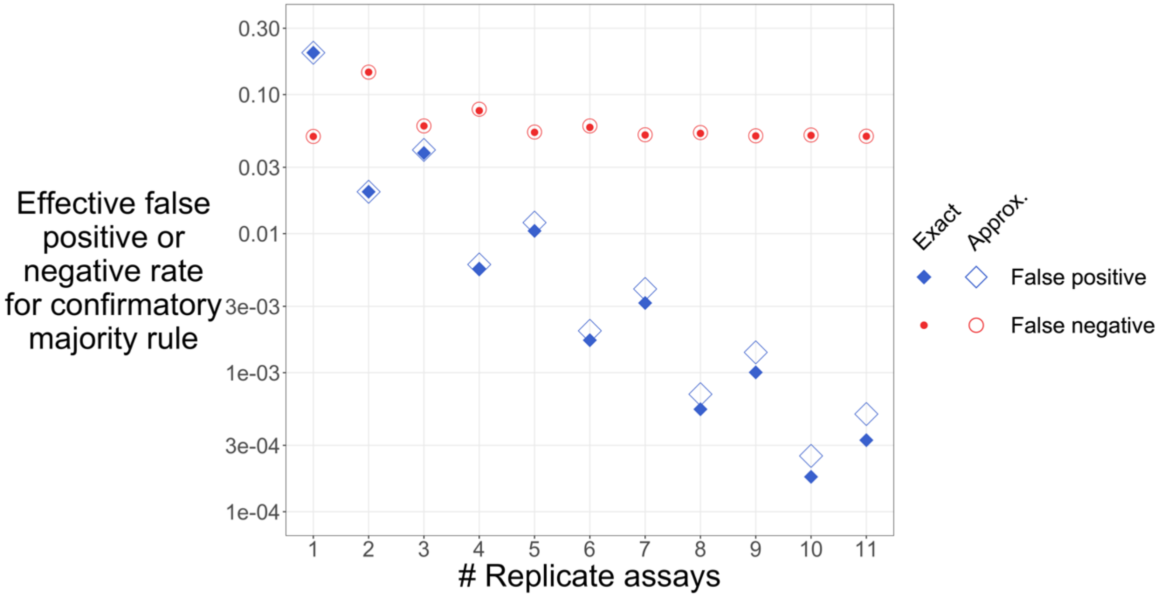

Figure S2. Adequacy of approximation for confirmatory majority rule sampling strategy (initial assay higher sensitivity and lower specificity than subsequent assays). Filled symbols show actual values (Eqs. 14 and 15, Methods); open symbols show approximations (Eqs. 16 and 17, Methods). **Parameters:** $FP_1 = 0.2$; $FN_1 = 0.05$; $FP_2 = FN_2 = 0.1$.
